# Perfectionism Contributes to Sleep-Wake State Discrepancy: The Mediating Role of Pre-Sleep Arousal

**DOI:** 10.3390/brainsci16060600

**Published:** 2026-05-31

**Authors:** Susie Y. Oh, Christian L. Nicholas, Lisa J. Phillips, David Cunnington, Maya T. Schenker, Cameron J. Patrick, Daniel Windred, Hailey Meaklim

**Affiliations:** 1 Melbourne School of Psychological Sciences, The University of Melbourne, Parkville, VIC 3010, Australia; ohsy@student.unimelb.edu.au (S.Y.O.); cln@unimelb.edu.au (C.L.N.); lisajp@unimelb.edu.au (L.J.P.); m.schenker@unimelb.edu.au (M.T.S.); 2 Monash University Healthy Sleep Clinic, Turner Institute for Brain and Mental Health, Monash University, Clayton, VIC 3168, Australia; 3 Thompson Institute, University of the Sunshine Coast, Birtinya, QLD 4575, Australia; 4 Statistical Consulting Centre, School of Mathematics and Statistics, The University of Melbourne, Parkville, VIC 3010, Australia; cameron.patrick@unimelb.edu.au; 5 Flinders Health and Medical Research Institute (FHMRI): Flagship Sleep Health Program, Flinders University, Adelaide, SA 5042, Australia

**Keywords:** perfectionism, sleep-wake state discrepancy, sleep misperception, insomnia, actigraphy, sleep health

## Abstract

**Objectives:** Sleep-wake state discrepancy, the discrepancy between self-reported and objective sleep measures, is commonly experienced in poor sleep and insomnia. While perfectionism is implicated in insomnia, its relationship to sleep-wake state discrepancy has not been investigated. This study aimed to assess the association between sleep-wake state discrepancy and perfectionism and explore whether dysfunctional sleep beliefs and pre-sleep arousal mediate that relationship. **Methods:** Sixty adult participants from community and clinical populations were conveniently sampled (85% females, mean age 30.28 ± 11.13 years, 38% with insomnia symptoms). Sleep-wake state discrepancy measures were calculated using data from actigraphy and sleep diary collected over 14 days. The Frost Multidimensional Perfectionism Scale (FMPS), Hewitt–Flett Multidimensional Perfectionism Scale (HFMPS), Dysfunctional Beliefs about Sleep (DBAS), and Pre-sleep Arousal Scale (PSAS) were also collected. **Results:** High perfectionism levels were associated with high levels of sleep-wake state discrepancy. Concern over Mistakes and Doubts about Actions correlated with sleep onset latency discrepancy with small effects (*r* = 0.26 and 0.29, respectively). Doubts about Actions was associated with sleep onset latency discrepancy. Furthermore, pre-sleep arousal and cognitive pre-sleep arousal mediated relationships between sleep onset latency discrepancy and Concern over Mistakes and Doubts about Actions. **Conclusions:** Concern over Mistakes and Doubts about Actions relate to a poorer perception of sleep relative to objective sleep measures. During sleep onset, cognitive pre-sleep arousal appears to mediate relationships between perfectionism and sleep-wake state discrepancy. Therefore, perfectionism may be an important cognitive-emotional factor to consider when assessing and treating sleep-wake state discrepancy that commonly accompanies insomnia.

## 1. Introduction

Poor sleep is a common and burdensome condition. Insomnia symptoms reflecting poor sleep can include difficulties initiating and/or maintaining sleep and/or dissatisfaction with sleep quality. While insomnia disorder is a formal diagnostic category requiring persistent sleep difficulties, significant daytime impairment, and occurring at least three times per week for a minimum of three months [1], the focus of this paper is on the broader experience of insomnia symptoms that reflect poor sleep, given their significant prevalence and impact even in the absence of a formal diagnosis. For example, in an Australian general population survey, 50% reported at least one insomnia symptom of difficulties initiating or maintaining sleep, while only 23% met diagnostic criteria for insomnia disorder [2]. Insomnia symptoms adversely affect general health and mental well-being, costing Australia an estimated USD 13.3 billion in 2019–2020 in lost productivity and healthcare-related costs [3,4]. It is therefore critical to identify the behavioural, emotional, and cognitive processes that contribute to poor sleep quality and insomnia symptoms.

Sleep quality can be measured by subjective reports or objectively using tools, such as polysomnography (PSG) and actigraphy. Subjective reports of poor sleep and insomnia symptoms rely on an individual’s self-reported perception of sleep and daytime functioning. Objective measures include PSG, which relies on multiple signals to measure sleep (e.g., electroencephalogram, electromyography), and actigraphy, which uses an accelerometer to infer sleep from physical activity in a simple, portable wrist-worn device [5]. Despite the benefits of capturing one night of comprehensive sleep data via PSG, actigraphy offers significant cost advantages and facilitates the collection of multiple nights of ecological sleep data from participants’ natural sleep environment [6].

The outcomes from self-reported sleep measures can differ from those obtained via objective measurement. It is estimated that 8 to 66% of people with insomnia self-reported their sleep to be worse (i.e., reporting less sleep and more wakefulness during the same sleep opportunity) compared to objective sleep measures [7]. On the other hand, good sleepers have been found to exhibit the opposite pattern of overestimating their sleep compared to objective sleep measures [8]. The discrepancy in sleep estimation between self-reported and objective measures of sleep is referred to by various names: sleep-state misperception, sleep misestimation, paradoxical insomnia, and subjective-objective sleep discrepancy. The term sleep-wake state discrepancy is used in this manuscript, adopting a recent recommendation that highlighted the importance of maintaining neutrality in the study of this discrepancy without implying the individual as inaccurate or incorrect, since its underlying mechanisms remain unknown [9,10]. While sleep-wake state discrepancy is not always a symptom of poor sleep, it is found with both PSG and actigraphy studies across various insomnia population groups [8,11,12,13,14,15,16,17]. Furthermore, compared to normal estimators, under- and over-estimators of sleeping time were found to report a lower quality of life [18]. Therefore, it is important to further our understanding of sleep-wake state discrepancy and any factors that may worsen this phenomenon.

Cognitive-emotional factors play an important role in insomnia and sleep-wake state discrepancy. Personality traits, rumination, anxiety and pre-sleep arousal all influence an individual’s perception of sleep and wakefulness, as well as sleep duration [19,20,21,22,23,24,25,26]. Specifically, perfectionism is a cognitive-emotional personality construct that has been suggested as a predisposing and possible perpetuating factor for insomnia [27,28]. An individual with heightened perfectionism is broadly characterised by having unrelenting standards and being excessively critical of one’s own performance and behaviours [29,30]. Past research has identified specific facets of perfectionism, including Concern over Mistakes (reacting adversely to and perceiving mistakes as failures), Doubts about Actions (having doubts or feeling uncertain about one’s own behaviours) and Socially Prescribed Perfectionism (believing others expect one to be perfect), to be associated with both objective and self-reported markers of poor sleep [27,31,32,33,34,35,36,37,38,39,40,41,42,43,44,45,46,47,48,49,50]. Notably, our previous study showed that perfectionism relates more strongly to self-reported than objective markers of poor sleep, and those who self-reported higher levels of sleep onset latency and lower sleep efficiency also reported higher levels of perfectionism [44]. However, whether sleep-wake discrepancy exists in individuals with perfectionism and how perfectionism may play a role in sleep-wake state discrepancy remains unknown.

Poor sleep may be maintained by an interaction between cognitive sleep beliefs (e.g., “I must get eight hours of sleep to function properly”) and hyperarousal with perfectionism. Riemann et al. [51] indicated that hyperarousal is a key feature in cognitive and/or neurobiological-based insomnia models, some of which attempt to account for the phenomenon of sleep-wake state discrepancy [10,52,53,54,55,56,57,58]. Pre-sleep arousal, a form of hyperarousal, can occur as somatic and/or cognitive activation during the transition to sleep, and is disruptive to sleep initiation and maintenance [59]. Somatic or physiological arousals relate to physical sensations experienced from a racing heart, bodily tensions, jitteriness or nervousness, while cognitive arousals relate to worries or a racing mind that is difficult to control. Hyperarousal and pre-sleep cognitive arousal have been found to be associated with sleep-related metacognitive processes in insomnia, which may contribute to sleep-wake state discrepancy [10,60]. Furthermore, dysfunctional beliefs about sleep, worry, and attentional bias have been suggested to perpetuate insomnia and are linked to sleep-wake state discrepancy [10,61]. Given that perfectionism is associated with higher physiological arousals and that individuals with perfectionistic concerns can become anxious about their sleep performance and subsequent effects of poor sleep to drive cognitive and emotional arousals, perfectionism is theorised to be a risk factor for poor sleep and may contribute to sleep-wake state discrepancy [51,57,62,63].

Additionally, anxiety and stress, dysfunctional beliefs about sleep, counterfactual processing and arousability have been identified as mediators in the relationship between perfectionism and poor sleep [32,33,38,44,46,48,50]. In a recent network analysis study, Zhao et al. [64] found direct associations between insomnia severity, dysfunctional beliefs and attitudes about sleep and hyperarousal. Furthermore, hyperarousal was identified as a key node linking insomnia and perfectionism, whereby Concern over Mistakes, Doubt about Actions, Parental Expectations and Personal Standards were positively associated with hyperarousal. In another study, it was suggested that potential interaction effects between perfectionism and pre-sleep arousal perpetuated insomnia symptoms based on the observation that individuals with high Concern over Mistakes and Doubts about Actions experienced poorer sleep on days of higher pre-sleep arousal [65]. To date, no study has explored whether and how perfectionism relates to hyperarousal or dysfunctional sleep beliefs and the impact this may have on sleep-wake state discrepancy.

This study extends our previous work, which found stronger relationships between perfectionism and self-reported sleep measures compared to objective sleep measures [44]. We aim to investigate whether sleep-wake state discrepancy plays a role in the relationships between perfectionism and poor sleep by (1) exploring the relationships between dimensions of perfectionism and measures of sleep-wake state discrepancy, and (2) investigating whether dysfunctional sleep beliefs and attitudes and pre-sleep arousal mediate the relationships between perfectionism and sleep-wake state discrepancy. No a priori hypothesis was made, given a lack of past research findings, and these aims are considered explorative.

## 2. Materials and Methods

### 2.1. Participants

Participants, aged 18 years and above, were recruited from the University of Melbourne’s Research Experience Program, through social media and word of mouth or referred from the Melbourne Sleep Disorder Centre by their sleep physician. Participants from the Research Experience Program were undergraduate psychology students who responded to an online advertisement at the University of Melbourne, Australia, to receive course credit for participation in the screening survey. Potential participants from all recruitment pathways were assessed for study eligibility by completing an online screening survey via Qualtrics (https://www.qualtrics.com, URL accessed on 09 January 2021). A diagnosis of clinical insomnia was not required for participation, but participants were required to self-report levels of insomnia symptomology using the Insomnia Severity Index (ISI) [66]. Therefore, the final sample consisted of participants from both clinical and general populations. Participants who reported the following were excluded: (a) current diagnosis of mental illness; (b) central nervous disorder; (c) prior head injury; (d) shift work, defined as paid employment with rotating shifts or including work hours outside of 7:00 AM to 6:00 PM, or starting work before 7:00 AM or after 2:00 PM; (e) symptoms consistent with sleep disorders other than insomnia (e.g., restless leg syndrome, obstructive sleep apnoea).

Initially, 1132 screening responses were obtained. After excluding duplicate or incomplete responses and those who declined to proceed with further study participation, 243 eligible participants were contacted for further study participation. Sixty-five participants agreed and participated in the two-week actigraphy sleep study, which resulted in a final sample of 60 after excluding participants with actigraphy recording errors (i.e., due to non-wear and/or data corruption) and/or incomplete survey responses. In the final sample, 7% were recruited from Melbourne Sleep Disorder Centre, 35% from the Research Experience Program, and 58% from social media or by word of mouth. Objective and self-reported sleep data from this current sample were previously analysed and published [44]. Using the same sample, the current study extends this work by investigating sleep-wake state discrepancy.

Informed written consent was obtained online from all participants. Upon completing the actigraphy sleep study, each participant received a $25 online gift voucher. The Human Research Ethics Committee at the University of Melbourne granted approval of this research study (HREC ID 1955374).

### 2.2. Sleep and Sleep-Related Measures

Collection of both self-reported and objective sleep measures occurred throughout the 14-day actigraphy study. The Pittsburgh Sleep Diary (PSD) [67] was delivered to participants’ smartphones daily via the Smartphone Ecological Momentary Assessment app (SEMA3) [68]. The PSD facilitated calculation of self-reported total sleep time (TST), sleep onset latency (SOL) and wake after sleep onset (WASO) by capturing daily self-reports of bedtime, lights out time, minutes to sleep, waketime, etc. To measure objective sleep, participants wore GENEActiv devices (Activinsights, Cambridge, UK) on their non-dominant wrist, recording accelerometer data at 40 Hz. GENEActive PC Software (version 3.3) was used to extract the accelerometer data before being processed with GGIR (version 2.6.0) using R (version 4.0.5). Sleep recordings were extracted based on T3A10 GGIR parameters, which detected sleep by the absence of arm angle changes above 10-degrees for at least 3 min. This setting was chosen over its default T5A5 setting because GGIR’s validation against polysomnography suggested that a reduced time window and increased angle threshold improved sensitivity in sleep detection [69]. Sleep recordings were evaluated and used to calculate objective TST and WASO. Objective SOL was calculated as the difference between participants’ self-reported lights-out time and GGIR-defined sleep onset time.

SWSD calculations. Daily sleep-wake state discrepancies were calculated as the differences between self-reported and objective measures of SOL, WASO and TST, as these arithmetic differences are most commonly used as measures of sleep discrepancy [70]. Higher discrepancy values indicated higher self-reported estimates of each sleep measure compared to objective sleep. After adjusting for missing or unusable data, the score for each sleep-wake state discrepancy variable for each participant was calculated as an average over the number of nights where usable data were recorded over the 14 days.

Insomnia Severity Index. At the end of the 14-day actigraphy sleep study, participants completed the Insomnia Severity Index (ISI) [66], which is a self-reported questionnaire measuring the nature, severity and impact of insomnia over the past two weeks. The ISI has 7 items, which are rated on a 5-point Likert scale from 0 (no problem) to 4 (very severe problem), with a total score ranging from 0 to 28. It has a good sensitivity of 86.1% and specificity at 87.7% for identifying insomnia in a community sample using a cut-off score of 10, and excellent internal consistency with Cronbach alphas of 0.90 and 0.91 for community and clinical samples, respectively [71].

Dysfunctional Beliefs and Attitudes about Sleep Scale and Pre-Sleep Arousal Scale. Other sleep-related measures collected at the start of the actigraphy sleep study period were the abbreviated version of the Dysfunctional Beliefs and Attitudes about Sleep Scale (DBAS) [61] and the Pre-Sleep Arousal Scale (PSAS) [59]. The DBAS consists of 16 items assessing the degree of dysfunctional sleep/insomnia-related thoughts, where each item is rated on a Likert scale from 0 (strongly disagree) to 10 (strongly agree). The DBAS covers four subscales of sleep-related thoughts that include two items on expectations about sleep (DBAS-E), six items on worries about sleep (DBAS-W), five items on consequences of insomnia (DBAS-C) and three items on beliefs about sleep medication (DBAS-M). All its subscales and total score range from 0 to 10 (average across the items), with a higher score indicating a higher level of dysfunctional beliefs and attitudes about sleep/insomnia. The DBAS is both internally consistent and stable over time, with Cronbach alphas of 0.77 in the clinical sample and 0.79 in the research sample, and a test-retest correlation coefficient of 0.83 [61]. The PSAS measures the level of arousal experienced at bedtime when attempting to initiate sleep. It includes two subscales consisting of eight somatic arousal (PSAS-S) symptoms and eight cognitive arousal (PSAS-C) symptoms. Each item is rated on a 5-point Likert scale from 1 (not at all) to 5 (extremely), giving rise to subscale scores ranging from 8 to 40, where higher scores indicate higher levels of arousal before falling asleep. The PSAS has good internal consistency with Cronbach’s alphas of PSAS-S and PSAS-C, ranging from 0.79 to 0.84 and 0.67 to 0.88 in college students, normal sleepers and insomniacs, respectively; as well as good temporal reliability with test-retest correlations of 0.76 for PSAS-S and 0.72 for PSAS-C [59].

### 2.3. Perfectionism Measures

The Frost Multidimensional Perfectionism Scale (FMPS) [29] and Hewitt–Flett Multidimensional Perfectionism Scale (HFMPS) [30] were used to assess the multidimensions of perfectionism. The FMPS has 35 items, which are rated on a Likert scale from 1 (disagree strongly) to 5 (agree strongly). These items form 6 subscales: Concern over Mistakes (reacting adversely to and perceiving mistakes as failures), Doubts about Actions (having uncertainty about one’s performance), Parental Expectations (perceiving parents to set high standards for oneself), Parental Criticism (perceiving parents as being highly critical of oneself), Personal Standards (having high self-evaluation standards) and Organisation (preferring order and structure). Refer to Table 1 for the score range of each subscale and total FMPS, which excludes the Organisation subscale. Higher scores indicate higher levels of perfectionism. Each subscale has good internal consistency with Cronbach’s alphas and reliability coefficients ranging from 0.77 to 0.93 and 0.77 to 0.94, respectively [29]. The HFMPS has 45 items, which are rated on a Likert scale from 1 (disagree) to 7 (agree), and three subscales: Self-oriented Perfectionism (expecting oneself to have high standards), Other-oriented Perfectionism (expecting others to have high standards) and Socially Prescribed Perfectionism (perceiving others to set high standards for oneself). Each subscale has a score range of 7 to 105, with higher levels of perfectionism indicated by higher scores. Each subscale has good internal consistency with Cronbach’s alphas ranging from 0.74 to 0.88 [30].

### 2.4. Study Protocol

After initial screening and prior to beginning the actigraphy study, the lead researcher (S.O.) met with each participant either in-person or online via Zoom (https://zoom.au, URL accessed on 21 April 2021). During this meeting, participants completed the FMPS, HFMPS, DBAS and PSAS; and were set up with or sent via post (prior to the online meeting) an actigraphy watch and given explanations about the actigraphy watch and study protocol. The accelerometer was worn by participants on their non-dominant wrist 24 h a day over the 14-day actigraphy study period. Removal of the accelerometer was allowed during swimming, contact sports and/or to meet work requirements. Over the 14 days, participants received daily notifications and reminders via SEMA3 within specific data collection input timeframes to fill in the PSD (e.g., participants were prompted and allowed to fill in the morning diary between 7:30 AM and 1:30 PM and the bedtime diary between 8:30 PM and 12:30 AM). At the end of the actigraphy study period, participants completed the ISI, were debriefed and thanked in a meeting, and received remuneration.

### 2.5. Statistical Analysis

All analyses were conducted using the statistical software package R (version 4.3.0). Due to the exploratory nature of this study, the relationships between perfectionism and sleep were assessed by correlation, regression, and mediation analyses. Firstly, the associations between perfectionism and sleep-wake state discrepancy were analysed using Pearson correlations, which were deemed to have a small effect at 0.10, a medium effect at 0.30, or a large effect at 0.50 [72]. Prior to further analysis, sleep-wake state discrepancy measures SOL_D (discrepancy in sleep onset latency), WASO_D (discrepancy in wake after sleep onset) and TST_D (discrepancy in total sleep time), which were derived from diary-actigraphy differences (sleep diary minus actigraphy) were visually inspected. Overall, model assumptions of normality were met.

A series of regression models was conducted for each combination of perfectionism variable (FMPS-total, HFMPS-total and subscale scores as independent variables) and sleep-wake state discrepancy measure (SOL_D, WASO_D, TST_D) as dependent variable. Age and gender were included as covariates. Using DBAS and PSAS and subscale scores as mediators, a series of mediation analyses were conducted for each selected combination of perfectionism subscale (Concern over Mistakes, Doubts about Actions) and each sleep-wake state discrepancy measure (SOL_D, WASO_D and TST_D). As per O’Rourke and MacKinnon [73], mediation analyses were conducted despite the absence of a significant total effect. The alpha level for the two-tailed analyses was set at *p* < 0.05.

## 3. Results

### 3.1. Descriptive Statistics

**Figure 1:**
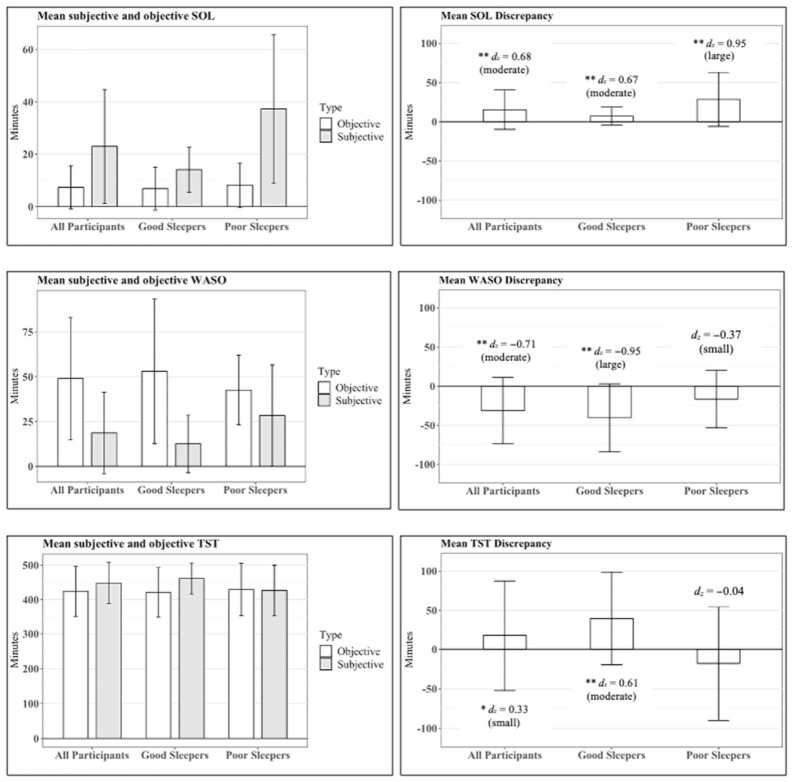
Sleep-wake state discrepancy measures across groups (mean and standard deviation). Notes. SOL = sleep onset latency; WASO = wake after sleep onset; TST = total sleep time. In discrepancy measures, a positive score denotes overestimation while a negative score denotes underestimation. Vertical lines depict standard deviation. Asterisk indicates a significant difference between self-reported and objective measures of sleep parameters, with Cohen’s d_z_ effect size presented. * *p* < 0.05, ** *p* < 0.01.

Sixty participants were aged 18–66 years (*M* = 30.28, *SD* = 11.13), with 85.0% identifying as female. Means and standard deviations of FMPS, HFMPS, DBAS and PSAS scores are presented in Table 1. Participants’ ISI scores ranged from 0 to 25 (*M* = 7.48, *SD* = 5.92), with 38.3% (*n* = 23) classed as a ‘poor sleeper’ based on an ISI score above 9, which classifies insomnia cases in community samples [71]. For the remainder of this paper, a ‘poor sleeper’ refers to participants with insomnia symptoms reporting an ISI score of greater than 9. Mean and standard deviation of sleep and discrepancy measures for the total sample, poor sleepers and good sleepers are presented in Figure 1.

Results in Table 2 show that, compared to actigraphy, participants classified as poor sleepers self-reported worse sleep than good sleepers. First, poor sleepers reported significantly higher SOL than good sleepers (*p* < 0.01) despite no significant difference in their actigraphy-derived SOL. Second, good sleepers, but not poor sleepers, significantly underestimated WASO compared to actigraphy (*p* < 0.01), even though their objective measures of WASO were not statistically different. Third, good sleepers reported significantly lower WASO than poor sleepers (*p* = 0.020). Therefore, good sleepers perceived better sleep with much less WASO across the night than was measured by actigraphy and when compared to poor sleepers, even though their objective WASO was not different to that of poor sleepers. Furthermore, good sleepers overestimated TST relative to actigraphy (*p* < 0.01) and reported higher TST (*p* = 0.049) despite having no difference in objective TST compared to poor sleepers. Given the lack of power, no further analysis was conducted on group differences in sleep discrepancy measures. Mean and standard deviation of self-reported and objective sleep parameters for all participants as a group can be found in the Supplementary Material Table S1.

### 3.2. Correlation and Regression Analyses of Sleep-Wake State Discrepancy Measures and Perfectionism

Results in Table 3 and Table 4 show significant positive correlations were found between SOL_D (discrepancy in sleep onset latency) and Concern over Mistakes (*r* = 0.26, *p* = 0.041) and Doubts about Actions (*r* = 0.29, *p* = 0.023), which were considered to have small-to-medium effects [72]. However, only Doubts about Actions was associated with sleep onset discrepancy in regression analysis, as no other perfectionism scale or subscales were significant predictors of the various sleep-wake state discrepancy measures.

### 3.3. Mediation Analyses Using DBAS and PSAS as Mediators in the Relationships Between Sleep Discrepancy Measures and Selected Measures of Perfectionism

**Figure 2:**
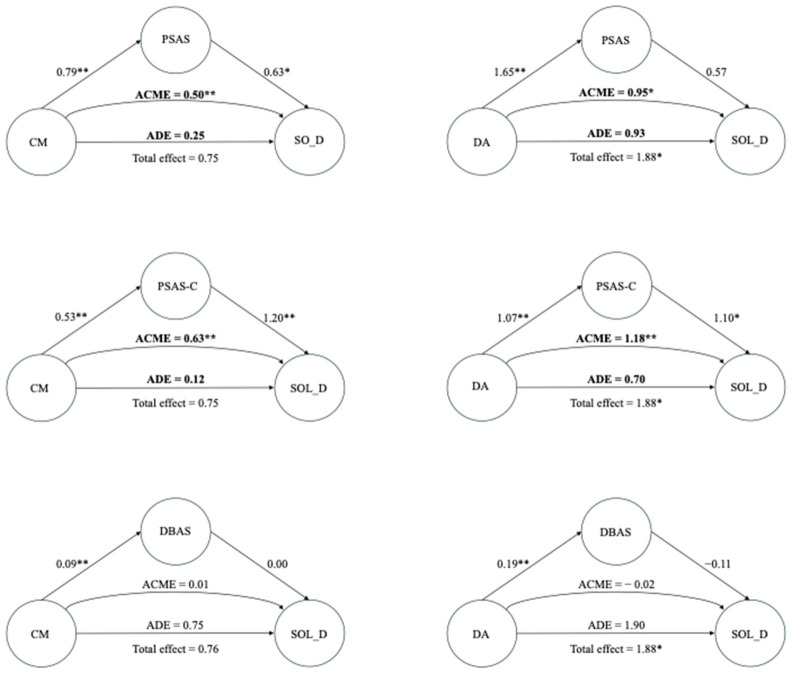
Results from mediation analyses using PSAS, PSAS Cognitive arousal subscale and DBAS as a mediator in the relationship between sleep onset latency discrepancy and Concern over Mistakes and Doubts about Actions subscales of perfectionism (*n* = 60, simulations = 10,000). Notes. ACME = average causal mediation effects; ADE = average direct effects; CM = Concerns over Mistakes; DA = Doubts about Actions; PSAS = Pre-Sleep Arousal Scale; PSAS-C = Pre-Sleep Arousal Scale Cognitive arousal subscale; DBAS = Dysfunctional Beliefs and Attitudes about Sleep Scale; SOL_D = discrepancy in sleep onset latency. * *p* < 0.05, ** *p* < 0.01.

Results from the mediation analyses largely mirrored the results from the regression analyses. None of the mediation analyses using DBAS, DBAS subscales, or the PSAS somatic subscale as mediators were significant (see Tables S2–S9 in Supplementary Material). Given results relating to SOL_D are in line with sleep-wake state discrepancy typically found in insomnia; results relating to SOL_D are presented here. All mediation analysis results relating to WASO_D and TST_D can be found in Tables S2–S9 in the Supplementary Material. As shown in Figure 2, while the total effect of Concern over Mistakes on SOL_D was not significant, the mediation effect of PSAS and PSAS-Cognition on that relationship was significant. An increase in Concern over Mistakes is associated with a larger discrepancy in SOL (higher perceived SOL relative to objective SOL) via PSAS and PSAS-Cognition as mediators. Similarly, the relationship between Doubts about Actions and SOL_D was mediated by PSAS and PSAS-Cognition. Included in Figure 2 for comparison, the mediation analyses using DBAS as a mediator in the relationships between Concern over Mistakes and Doubts about Actions with SOL_D were not significant. Considering the number of analyses conducted in the current study, which could increase the risk of Type 1 error, results should be interpreted with caution.

## 4. Discussion

This study explored the relationships between dimensions of perfectionism and sleep-wake state discrepancy, as measured by self-reported and actigraphy-derived objective sleep measures. It was explored whether these relationships were mediated by dysfunctional sleep beliefs and attitudes and pre-sleep arousal. Results showed that individuals who reported higher levels of perfectionism also demonstrated higher levels of sleep-wake state discrepancy. Specifically, those who reported higher levels of Concern over Mistakes and Doubts about Actions also reported higher discrepancy in sleep onset latency. Furthermore, Doubts about Actions was a significant predictor of sleep onset latency discrepancy, and that relationship was mediated by pre-sleep arousal and cognitive pre-sleep arousal. Pre-sleep arousal and cognitive pre-sleep arousal also mediated the relationship between Concern over Mistakes and sleep onset latency discrepancy. Overall, these results suggest certain aspects of perfectionism (i.e., Concern over Mistakes and Doubts about Actions) relate to a poorer perception of sleep relative to objective sleep measures. Exploratory analyses suggest that cognitive pre-sleep arousal may mediate this relationship and is associated with sleep-wake state discrepancy during sleep onset.

Compared to actigraphy, poor sleepers in the current study perceived their sleep to be worse than that of good sleepers despite having similar or better objective sleep measures. This is consistent with the results of previous studies examining the sleep-wake state discrepancy in insomnia patients and healthy controls using actigraphy [8,12,16,74]. The current findings suggest that sleep-wake state discrepancy may play a role in the relationship between perfectionism and poor sleep. The correlations between perfectionism dimensions of Concern over Mistakes and Doubts about Actions with discrepancy in sleep onset latency, as well as the significant association between Doubts about Actions and sleep onset latency discrepancy, are consistent with past research findings that identified these facets of perfectionism to be associated with markers of poor sleep and insomnia [27,31,32,33,35,38,39,44,46,47,48,49,50]. It also suggests sleep-wake state discrepancy may account for the stronger relationship that perfectionism has with self-reported compared to objective markers of poor sleep, as found in our previous study [44]. Given that sleep-wake state discrepancy is commonly experienced by people with insomnia, this provides a new line of preliminary evidence to support the theory that perfectionism may serve as a predisposing and potential perpetuating factor for insomnia [7,27,28].

We observed self-reported overestimation of sleep onset latency and total sleep time, and underestimation of wake after sleep onset relative to actigraphy measures. This is consistent with findings from a large community sample of women in the United States by Lehrer et al. [75]. These findings may appear to contradict past research indicating that patients with insomnia tend to underestimate total sleep time relative to objective measures of sleep using actigraphy [10,76,77]. However, the difference may be accounted for by our current predominant community-based sample compared to clinical samples used in previous studies. This finding is also consistent with past demographic studies showing that sleep tends to be overestimated in the general population [18,20,78,79]. Furthermore, some studies have also reported the underestimation of self-reported WASO relative to objective WASO using actigraphy and polysomnography [11,12,16]. Given that actigraphy proxies sleep via a lack of body movement, the detection of minor body movement during sleep as wake could have also contributed to the overestimation of WASO relative to self-report [69,80,81].

Although both good and poor sleepers in our sample underestimated wake after sleep onset, the difference between self-reported and actigraphy-derived wake after sleep onset was not significant in poor sleepers, but it was significant in good sleepers. This is consistent with the finding that people with insomnia perceive their wake after sleep onset to be consistent with actigraphy-derived wake after sleep onset [8]. It also aligns with the recommendation that, unlike actigraphy measures of sleep onset latency and total sleep time, actigraphy wake after sleep onset is considered imprecise compared to polysomnography in assessing and treating adult insomnia [82]. Given the above and that self-reported total sleep time was derived using wake after sleep onset, we focused our attention on sleep onset latency discrepancy.

Pre-sleep arousal, specifically cognitive pre-sleep arousal, mediated the relationship between sleep onset latency discrepancy and Doubts about Actions, as well as Concern over Mistakes. This current finding parallels past research that identified arousability and counterfactual processing as mediators in the relationship between perfectionism and poor sleep [38,46,48]. It is also consistent with recent research supporting the role of hyperarousal and pre-sleep arousal in insomnia. Zhao et al. [64] identified hyperarousal as the link between insomnia and the perfectionism dimensions of Concern over Mistakes and Doubts about Actions. Similarly, Kuskens et al. [65] found that those with higher Concern over Mistakes and Doubts about Actions experienced worse sleep on days of higher pre-sleep arousal than on days of lower pre-sleep arousal. This study takes the literature further by potentially identifying sleep-wake state discrepancy as a plausible symptom of poor sleep experienced by those who have high levels of perfectionism and experience hyperarousal in the pre-sleep period.

Individuals who are highly perfectionistic are thought to experience higher arousal in the period before sleep, which may contribute to a delay in sleep onset [27,47]. One plausible explanation for the current findings may be that on nights when individuals are aroused due to being excessively concerned about mistakes or doubting their own actions that might have occurred during the day or in the past, sleep onset can become delayed due to rumination or worry. As such, the automaticity of sleep normalcy becomes disrupted, which would be consistent with the attention-intention-effort pathway model of insomnia [57]. Given that self-reported sleep is assessed the next morning in studies of sleep-wake discrepancy, those who excessively doubt their own actions may subsequently doubt their ability to initiate sleep, leading them to question how long it took for them to fall asleep and/or even question their time asleep and mistaking it as wake, leading to an overestimation of sleep onset latency. This is consistent with time overestimation and perceiving sleep as wake in explaining sleep-wake state discrepancy when experiencing higher cognitive arousal, which were suggested to be related to worry, selective attention and monitoring [10,26,83]. Furthermore, this ties in with past research that found worry, anxiety and rumination to be significantly associated with Concern over Mistakes and Doubts about Actions [84,85,86] The current findings, therefore, add exploratory evidence to suggest pre-sleep arousal as an indirect effect through which perfectionism may influence the cognitive and emotional interpretations of sleep experiences associated with sleep-wake state discrepancy, which can lead to perception of poor sleep.

Notably, the current study did not find evidence to support the mediating role of dysfunctional sleep cognitions in the relationship between perfectionism and sleep-wake state discrepancy. This is inconsistent with a previous finding of the mediating role of dysfunctional sleep cognitions between insomnia symptoms and Doubts about Actions using a similar but much larger general population sample by Akram et al. [33]. It is plausible that the current sample size was too small to detect the effects or that sleep-wake state discrepancy is not a symptom of insomnia that is affected by these dysfunctional sleep cognitions. However, our current study is consistent with the network analysis findings in a recent study of young adults by Zhao et al. [64]. The authors found only hyperarousal, but not dysfunctional sleep cognitions, to be a key node linking insomnia and perfectionism despite finding direct associations between insomnia severity, dysfunctional beliefs and attitudes about sleep and hyperarousal. The role, if any, that dysfunctional sleep cognitions play in the relationships between perfectionism and sleep-wake state discrepancy and insomnia remains unclear.

### 4.1. Theoretical and Clinical Implications

Results from the current study provide new exploratory evidence supporting prior theoretical conceptualisations and empirical literature suggesting that perfectionism is a contributing factor in poor sleep and insomnia. Specifically, pre-sleep arousal was identified as an indirect effect in which perfectionism may lead to sleep-wake state discrepancy. It is not uncommon for people with insomnia to report their sleep to be worse than objective sleep measures obtained simultaneously [7]. This study has identified sleep-wake state discrepancy to be associated with perfectionism via the mediating effect of pre-sleep arousal. Together, these provide insight into the theoretical conceptualisation of how perfectionism may serve as a plausible predisposing and perhaps perpetuating risk factor for poor sleep in line with cognitive models of insomnia [7,27,28,47].

Although this study was conducted primarily on a community-based sample, it may provide some important clinical insights for insomnia management. It is possible that the current findings could have stronger effects in a clinical sample. Nevertheless, the following discussion on clinical insights is speculative, given the exploratory nature of the current study. It may be important for clinicians to consider possible traits of perfectionism, particularly Concern over Mistakes and Doubts about Actions, in addition to initial diagnostic indications of prolonged self-reported sleep onset latency when assessing the nature of their patients’ sleep perceptions. The specific subscales of the FMPS can be easily incorporated into clinical practice to screen for such traits of perfectionism. Furthermore, clinicians should exercise caution in relying on self-reported sleep measures and consider supplementing with objective measures in the assessment and monitoring of sleep in this population group, particularly where sleep-state wake discrepancy may be contributing to the complaints of poor sleep. As suggested by Fernandez-Mendoza et al. [20], different sleep phenotypes may respond better to different treatment approaches. The current results, if confirmed with a clinical sample in the future, suggest that in individuals who present with significant sleep-wake state discrepancy and high levels of perfectionism, it may be more appropriate to implement Cognitive Behavioural Therapy for Insomnia and Acceptance and Commitment Therapy for Insomnia than relying on sleep medication to reduce physiological hyperarousal and/or increase sleep duration. For example, providing psychoeducation to normalise sleep-wake state discrepancy, incorporating behavioural strategies, such as avoiding clock checking and introducing acceptance, mindfulness practice and relaxation training to reduce hyperarousal. Such strategies may also be more relevant than addressing dysfunctional beliefs and attitudes about sleep in the treatment of sleep-wake state discrepancy for this population group if the current findings hold true with a clinical sample. Lastly, treating perfectionistic concerns of making mistakes and doubts about actions, particularly if they relate to sleep, may also be helpful in therapy. Such modifications to current standard treatment therapy will need to be tested in future research.

### 4.2. Strengths, Limitations and Future Directions

To our best knowledge, this is the first study to explore relationships between perfectionism and sleep-wake state discrepancy relating to poor sleep. Although actigraphy provided good ecological sleep data for this study, it lacks the capacity to capture other physiological and neurobiological markers of sleep, such as electrical brain activity, increased heart rate variability, muscle responses and cortical arousals, which have been suggested to contribute to sleep-wake state discrepancy [10]. Actigraphy may have contributed to a key limitation in the current study, as self-reported lights-out time was used to partially derive objective sleep onset latency, which may be partly attributable to assessment artefact, to reduce the accuracy of the actual incongruence between self-reported and objective discrepancy [70]. While GeneActiv actigraphy is capable of estimating lights-out time based on the lack of movement and light, its accuracy is variable when relying on actigraphy alone, e.g., reading in bed under dim light while wearing long sleeve top, can be detected as sleep [87]. Although the processing of GeneActiv actigraphy was found to be comparable with and without a sleep diary, a sleep diary is generally recommended when using actigraphy [88,89]. Consequently, the current study adopted the use of a sleep diary to identify lights-out in an attempt to improve the accuracy of deriving objective sleep onset latency. Therefore, replicating this study using EEG-derived sleep estimates is needed to confirm the effects found here.

In a recent scoping review, Walton et al. [70] identified alternate ways of computing sleep discrepancy measures other than the arithmetic difference method used in the current study. For example, the Sleep Perception Index, as demonstrated by Lecci et al. [90] and Cini et al. [91], allows for comparisons between sleep metrics and identification of patterns in discrepancy across sleep metrics. While no comparison between sleep metrics was made in the current study and arithmetic differences are a commonly used method of calculating discrepancy measures [70] (e.g., Bensen-Boakes et al. [92], Naito et al. [93], Spina et al. [94], Bensen-Boakes et al. [95], Moulder et al. [96]), the Sleep Perception Index may be considered in future research. Until a consensus on sleep discrepancy measures and derivation is reached in the field, future studies should consider utilising other methods of sleep monitoring, such as PSG, and different ways of deriving sleep discrepancy measures to explore if these factors play a role in the relationships between perfectionism and insomnia.

While the nature of this study was exploratory, the relatively small sample size with numerous analyses may increase the risk of type 1 error, and thus the results should be interpreted with caution. This will need further investigation in larger studies with prior hypotheses. Another limitation of this study is that the resulting generalisations are based on a sample that consists of a small clinical subsample and is predominantly female. While gender was included as a covariate in the analysis, and insomnia is more prevalent in females, cautious generalisation of findings is warranted [97]. The current findings were considered to have small effects and were found using a general population sample that included both good and poor sleepers, which could have contributed to the conservative findings. Therefore, it is possible that stronger effects may be found using clinical samples. Although initial descriptive data analysis suggests differences exist between good and poor sleepers, between-group analysis was not conducted due to a lack of power. It will be ideal to recruit a larger sample, include both clinical and control samples and conduct between-group analysis in future studies. The cross-sectional data also do not allow for causality to be determined, so future longitudinal studies are recommended. Another limitation of the current study relates to the averaging of multiple nights’ data, which assumes consistency across nights in patterns of sleep over/underestimation [70]. Therefore, future studies can focus on intra-individual differences across nights in these relationships to test whether daily fluctuations in levels of perfectionism impact on sleep and/or sleep-wake state discrepancy; and whether and how pre-sleep arousal plays a part in those relationships if they exist.

## 5. Conclusions

This is the first study to explore perfectionism as a possible factor in sleep-wake discrepancy in relation to markers of poor sleep. The current study found that perfectionism facets of high levels of Concern about Mistakes and Doubts about Actions may increase one’s vulnerability to experiencing sleep-wake state discrepancy, and that relationship may be facilitated by heightened levels of pre-sleep arousal. These findings suggest perfectionism may be an important consideration in the assessment and treatment of poor sleep.

## Figures and Tables

**Table 1:** Mean scores, standard deviations, and sample range for measures of perfectionism, dysfunctional sleep beliefs and attitudes, and pre-sleep arousal for all participants.

	*M*	*SD*	Sample Range (Score Range)
FMPS	86.9	19.69	41–141 (29–145)
Concern over Mistakes	26.92	8.18	11–45 (9–45)
Doubts about Actions	11.87	3.77	5–20 (4–20)
Parental Criticism	9.37	4.04	4–20 (4–20)
Parental Expectation	13.2	4.54	6–25 (5–25)
Organisation	24.58	3.43	15–30 (6–30)
Personal Standards	25.58	5.65	9–35 (7–35)
HFMPS	177.2	29.85	100–238 (45–315)
Self-oriented Perfectionism	72.3	14.9	33–105 (15–105)
Other-oriented Perfectionism	52.48	13.36	23–81 (15–105)
Socially Prescribed Perfectionism	52.47	13.37	28–105 (15–105)
DBAS	4.68	1.91	0.44–10 (0–10)
DBAS-Expectations	6.46	2.70	0–10 (0–10)
DBAS-Worries	4.23	2.37	0–10 (0–10)
DBAS-Consequences	5.59	2.19	0.40–10 (0–10)
DBAS-Medication	2.88	2.18	0–10 (0–10)
PSAS	34.93	12.60	17–79 (16–80)
PSAS-Somatic	14.05	5.35	8–39 (8–40)
PSAS-Cognitive	20.88	8.32	8–10 (8–40)

Notes. FMPS, Frost Multidimensional Perfectionism Scale; HFMPS, Hewitt-Flett Multidimensional Perfectionism Scale; DBAS, Dysfunctional Beliefs and Attitudes about Sleep; PSAS, Pre-Sleep Arousal Scale.

**Table 2:** Mean scores and standard deviations of sleep measures by groups with within and between-group differences.

	Self-Reported	Objective	Within-Group Difference
	SOL_S	SOL_O	SOL (*t*-value)
Good sleepers	14.07 ± 8.58	6.84 ± 8.17	4.09 **
Poor sleepers	37.28 ± 28.29	8.20 ± 8.44	4.57 **
Between-group difference (*t*-value)	3.83 **	0.62	
	WASO_S	WASO_O	WASO (*t*-value)
Good sleepers	12.58 ± 16.05	53.04 ± 40.27	−5.76 **
Poor sleepers	28.40 ± 28.25	42.54 ± 19.41	−1.77
Between-group difference (*t*-value)	2.45 *	−1.35	
	TST_S	TST_O	TST (*t*-value)
Good sleepers	461.20 ± 44.96	421.07 ± 71.53	3.68 **
Poor sleepers	426.51 ± 73.40	429.38 ± 75.73	−0.19
Between-group difference (*t*-value)	−2.04 *	−0.43	

Notes. SOL = sleep onset latency; WASO = wake after sleep onset; TST = total sleep time. * *p* < 0.05, ** *p* < 0.01.

**Table 3:** Correlations between sleep discrepancy measures, perfectionism, sleep beliefs and attitude and pre-sleep arousal.

*n* = 60	1	2	3	4	5	6	7	8	9	10	11	12	13	14	15	16	17	18	19	20	21
1. SOL_D																					
2. WASO_D	0.32 *																				
3. TST_D	−0.66 **	−0.73 **																			
4. FMPS	0.23	0.01	−0.14																		
5. CM	0.26 *	0.01	−0.15	0.88 **																	
6. DA	0.29 *	0.02	−0.21	0.71 **	0.62 **																
7. PC	0.08	−0.06	−0.02	0.71 **	0.48 **	0.42 **															
8. PE	−0.05	−0.05	0.05	0.62 **	0.30 *	0.19	0.72 **														
9. O	0.01	0.03	0.03	0.09	0.07	0.08	−0.16	0.03													
10. PS	0.22	0.11	−0.18	0.74 **	0.62 **	0.44 **	0.20	0.29 *	0.26 *												
11. HFMPS	0.09	−0.01	−0.09	0.79 **	0.70 **	0.52 **	0.46 **	0.45 **	0.13	0.70 **											
12. SOP	0.14	−0.01	−0.09	0.72 **	0.67 **	0.53 **	0.25	0.23	0.29 *	0.82 **	0.76 **										
13. OOP	−0.13	0.08	−0.07	0.20	0.13	0.05	0.20	0.25	−0.08	0.13	0.56 **	0.02									
14. SPP	0.18	−0.09	−0.03	0.76 **	0.67 **	0.53 **	0.55 **	0.50 **	0.05	0.52 **	0.83 **	0.57 **	0.22								
15. DBAS	0.10	−0.03	0.06	0.47 **	0.39 **	0.36 **	0.34 **	0.33 *	0.32 *	0.31 *	0.42 **	0.37 **	0.03	0.49 **							
16. DBAS-E	−0.20	0.16	0.20	0.19	0.06	0.20	0.22	0.22	0.40 **	0.10	0.15	0.19	−0.01	0.13	0.60 **						
17. DBAS-W	0.13	0.04	−0.02	0.46 **	0.42 **	0.30 *	0.31 *	0.30 *	0.21	0.33 **	0.46 **	0.35 **	0.10	0.53 **	0.92 **	0.37 **					
18. DBAS-C	0.13	−0.09	0.09	0.41 **	0.33 *	0.43 **	0.29 *	0.23	0.30 *	0.29 *	0.33 *	0.33 **	0.02	0.34 **	0.86 **	0.57 **	0.66 **				
19. DBAS-M	0.12	0.06	0.02	0.33 **	0.29 *	0.19	0.25	0.30 *	0.21	0.18	0.27 *	0.24	−0.11	0.45 **	0.74 **	0.19	0.71 **	0.45 **			
20. PSAS	0.37 **	0.16	−0.32 *	0.51 **	0.54 **	0.51 **	0.22	0.13	0.01	0.39 **	0.48 **	0.49 **	−0.06	0.59 **	0.57 **	0.09	0.59 **	0.48 **	0.49 **		
21. PSAS-S	0.21	0.08	−0.19	0.40 **	0.42 **	0.41 **	0.28 *	0.12	−0.10	0.22	0.45 **	0.37 **	0.00	0.58 **	0.46 **	0.04	0.48 **	0.37 **	0.42 **	0.88 **	
22. PSAS-C	0.42 **	0.18	−0.36 **	0.51 **	0.54 **	0.51 **	0.16	0.13	0.08	0.44 **	0.44 **	0.50 **	−0.09	0.51 **	0.56 **	0.11	0.57 **	0.48 **	0.47 **	0.95 **	0.68 **

Notes. SOL_D = discrepancy in sleep onset latency; WASO_D = discrepancy in wake after sleep onset; TST_D = discrepancy in total sleep time; FMPS = Frost Multidimensional Perfectionism Scale; CM = Concern over Mistakes; DA = Doubts about Actions; PE = Parental Expectations; PC = Parental Criticism; O = Organisation; PS = Personal Standards; HFMPS = Hewitt–Flett Multidimensional Perfectionism Scale; SOP = Self-oriented Perfectionism; OOP = Other-oriented Perfectionism; SPP = Socially Prescribed Perfectionism; DBAS = Dysfunctional Beliefs and Attitudes about Sleep; DBAS-E = DBAS subscale Sleep Expectations; DBAS-W = DBAS subscale Worry about Sleep; DBAS-C = DBAS subscale Consequences of Insomnia; DBAS-M = DBAS subscale Medication; PSAS = Pre-sleep Arousal Scale; PSAS-S = Somatic subscale of the Pre-sleep Arousal Scale; PSAS-C = Cognitive subscale of the Pre-sleep Arousal Scale. * p < 0.05 ** p < 0.01.

**Table 4:** Regression coefficients between sleep discrepancy measures and perfectionism.

*n* = 60	SOL_D			WASO_D			TST_D		
	*β*	95% *CI*	*p*	*β*	95% *CI*	*p*	*β*	95% *CI*	*p*
FMPS	0.28	−0.06, 0.62	0.11	0.08	−0.50, 0.66	0.78	−0.51	−1.45, 0.44	0.29
CM	0.75	−0.06, 1.57	0.07	0.23	−1.19, 1.64	0.75	−1.36	−3.66, 0.94	0.24
DA	1.87	0.14, 3.61	0.03 *	0.42	−2.60, 3.45	0.78	−3.87	−8.74, 1.00	0.12
PC	0.44	−1.22, 2.09	0.60	−0.58	−3.36, 2.19	0.67	−0.29	−4.86, 4.28	0.90
PE	−0.40	−1.88, 1.09	0.59	−0.35	−2.84, 2.14	0.78	0.90	−3.20, 5.00	0.66
O	0.07	−1.93, 2.07	0.95	0.23	−3.13, 3.59	0.89	1.38	−4.13, 6.90	0.62
PS	0.97	−0.19, 2.13	0.10	0.83	−1.16, 2.81	0.41	−2.01	−5.25, 1.23	0.22
HFMPS	0.06	−0.17, 0.29	0.60	0.04	−0.35, 0.42	0.85	−0.27	−0.90, 0.36	0.39
SOP	0.21	−0.24, 0.66	0.35	0.21	−0.24, 0.66	0.35	−0.37	−1.62, 0.88	0.56
OOP	−0.28	−0.79, 0.23	0.28	0.35	−0.51, 1.21	0.41	−0.57	−1.98, 0.85	0.43
SPP	0.31	−0.20, 0.82	0.23	−0.18	−1.04, 0.69	0.68	−0.34	−1.77, 1.08	0.63

Notes. SOL_D = discrepancy in sleep onset latency; WASO_D = discrepancy in wake after sleep onset; TST_D = discrepancy in total sleep time; FMPS = Frost Multidimensional Perfectionism Scale; CM = Concern over Mistakes; DA = Doubts about Actions; PE = Parental Expectations; PC = Parental Criticism; O = Organisation; PS = Personal Standards; HFMPS = Hewitt–Flett Multidimensional Perfectionism Scale; SOP = Self-oriented Perfectionism; OOP = Other-oriented Perfectionism; SPP = Socially Prescribed Perfectionism. * *p* < 0.05.

## Data Availability

The data underlying this article cannot be shared publicly as it contains information that could compromise the privacy of research participants. De-identified data will be shared on a reasonable request to the corresponding author.

## Supplementary Materials

The following supporting information can be downloaded at: https://www.mdpi.com/article/10.3390/brainsci16060600/s1, Table S1: Mean scores and standard deviations for objective and subjective sleep measures; Table S2: Results from mediation analysis using PSAS as a mediator in the relationship between sleep discrepancy measures and selected subscales of perfectionism (*n* = 60, simulations = 10,000); Table S3: Results from mediation analysis using PSAS subscale Somatic (PSAS-C) as a mediator in the relationship between sleep discrepancy measures and selected subscales of perfectionism (*n* = 60, simulations = 10,000); Table S4: Results from mediation analysis using PSAS subscale Cognition as a mediator in the relationship between sleep discrepancy measures and selected subscales of perfectionism (*n* = 60, simulations = 10,000); Table S5: Results from mediation analysis using DBAS as a mediator in the relationship between sleep discrepancy measures and selected subscales of perfectionism (*n* = 60, simulations = 10,000); Table S6: Results from mediation analysis using DBAS subscale sleep Expectations (DBAS-E) as a mediator in the relationship between sleep discrepancy measures and selected subscales of perfectionism (*n* = 60, simulations = 10,000); Table S7: Results from mediation analysis using DBAS subscale Worry about sleep (DBAS-W) as a mediator in the relationship between sleep discrepancy measures and selected subscales of perfectionism (*n* = 60, simulations = 10,000); Table S8: Results from mediation analysis using DBAS subscale Consequences of insomnia (DBAS-C) as a mediator in the relationship between sleep discrepancy measures and selected subscales of perfectionism (*n* = 60, simulations = 10,000); Table S9: Results from mediation analysis using DBAS subscale Medication (DBAS-M) as a mediator in the relationship between sleep discrepancy measures and selected subscales of perfectionism (*n* = 60, simulations = 10,000).

## Abbreviations

CM	Concern over mistakes
DA	Doubts about actions
DBAS	Dysfunctional Beliefs and Attitudes about Sleep Scale
DBAS-C	Dysfunctional Beliefs and Attitudes about Sleep Scale—consequences of insomnia
DBAS-E	Dysfunctional Beliefs and Attitudes about Sleep Scale—sleep expectations
DBAS-M	Dysfunctional Beliefs and Attitudes about Sleep Scale—sleep medication
DBAS-W	Dysfunctional Beliefs and Attitudes about Sleep Scale—worries about sleep
FMPS	Frost Multidimensional Perfectionism Scale
HFMPS	Hewitt-Flett Multidimensional Perfectionism Scale
ISI	Insomnia Severity Index
O	Organisation
OOP	Other-oriented perfectionism
PC	Parental criticism
PE	Parental expectations
PS	Persona standards
PSAS	Pre-Sleep Arousal Scale
PSAS-C	Pre-Sleep Arousal Scale—cognitive arousal
PSAS-S	Pre-Sleep Arousal Scale—somatic arousal
PSD	The Pittsburgh Sleep Diary
PSG	polysomnography
SEMA3	Smartphone Ecological Momentary Assessment app
SOL	sleep onset latency
SOL_D	discrepancy in sleep onset latency
SOP	self-oriented perfectionism
SPP	socially prescribed perfectionism
TST	total sleep time
TST_D	discrepancy in total sleep time
WASO	wake after sleep onset
WASO_D	discrepancy in wake after sleep onset
