# Supplementary material for: Perfectionism Contributes to Sleep-Wake State Discrepancy: The Mediating Role of Pre-Sleep Arousal
Source: Brain Sci. 2026 May 31;16(6):600. doi: 10.3390/brainsci16060600 (PMC13296699; doi:10.3390/brainsci16060600)
Supplement: Supplementary file 1 [file brainsci-16-00600-s001.zip › brainsci-4309124-supplementary.pdf]

Supplementary Materials

Table S1. Mean scores and standard deviations for objective and subjective sleep measures

	Subjective		Objective		Paired t-Test		Effect Size	Discrepancy	
	M	SD	M	SD	t	P	Cohen's d_z	M	SD
SOL, mins	22.97	21.74	7.36	8.23	5.24	<.001**	0.68 (moderate)	15.29	25.21
WASO, mins	18.65	22.69	49.02	34.00	-5.50	<.001**	-0.71 (moderate)	31.44	42.37
TST, mins	447.90	59.43	424.30	72.65	2.58	.012*	0.33 (small)	17.55	69.79

Notes. SOL, sleep onset latency; WASO, wake after sleep onset; TST, total sleep time. $^*p < .05$ $^{**}p < .01$

Table S2. Results from mediation analysis using PSAS as a mediator in the relationship between sleep discrepancy measures and selected subscales of perfectionism ($n = 60$, simulations = 10,000)

Sleep Discrepancy	SOL_D			WASO_D			TST_D		
Perfectionism	Estimate	*95% CI*	*p*	Estimate	*95% CI*	*p*	Estimate	*95% CI*	*p*
CM									
- ACME	0.50	0.11, 1.01	.008**	0.63	-0.27, 1.72	.17	-1.57	-3.19, -0.33	.009**
- ADE	0.26	-0.58, 1.08	.55	-0.41	-2.49, 1.64	.70	0.19	-2.91, 3.34	.91
- Total effect	0.76	-0.03, 1.54	.06	0.22	-1.27, 1.63	.74	-1.38	-3.88, 1.28	.29
DA									
- ACME	0.95	0.12, 2.10	.020*	1.30	-0.35, 3.39	.13	-2.90	-6.01, -0.67	.006**
- ADE	0.93	-0.68, 2.51	.26	-0.89	-4.52, 2.74	.64	-0.95	-6.25, 4.48	.73
- Total effect	1.87	0.03, 1.99	.018*	0.42	-14.83, 15.28	.75	-3.84	-8.60, 1.11	.13

Notes. CM = Concern over Mistakes; DA = Doubts about Actions; ACME = average causal mediation effects; ADE = average direct effects. $^*p < .05$ $^{**}p < .01$.

Table S3. Results from mediation analysis using PSAS subscale Somatic (PSAS-C) as a mediator in the relationship between sleep discrepancy measures and selected subscales of perfectionism (*n* = 60, simulations = 10,000)

Sleep Discrepancy	SOL_D			WASO_D			TST_D		
Perfectionism	Estimate	*95% CI*	*p*	Estimate	*95% CI*	*p*	Estimate	*95%CI*	*p*
CM									
- ACME	0.43	-0.62, 1.61	.42	0.25	-0.59, 1.26	.57	-0.64	-2.11, 0.36	.23
- ADE	0.61	-.028, 1.53	.19	-0.01	-2.21, 2.00	1.00	-0.73	-3.84, 2.45	.65
- Total effect	1.04	.05, 2.08	.04*	0.23	-1.13, 1.69	.75	-1.37	-3.77, 1.21	.28
DA									
- ACME	0.26	-0.55, 1.24	.51	0.57	-1.14, 2.67	.51	-1.19	-4.00, 0.81	.25
- ADE	1.62	-0.15, 3.44	.07	-0.13	-3.76. 3.55	.95	-2.68	-8.37, 3.05	.36
- Total effect	1.88	0.33, 3.42	.02*	0.43	-2.28, 2.99	.73	-3.87	-8.47, 0.89	.11

Notes. CM = Concern over Mistakes; DA = Doubts about Actions; ACME = average causal mediation effects; ADE = average direct effects. *p < .05.

Table S4. Results from mediation analysis using PSAS subscale Cognition as a mediator in the relationship between sleep discrepancy measures and selected subscales of perfectionism (*n* = 60, simulations = 10,000)

Sleep Discrepancy	SOL_D			WASO_D			TST_D		
Perfectionism	Estimate	*95% CI*	*p*	*Estimate*	*95% CI*	*p*	Estimate	*95% CI*	*p*
CM									
- ACME	0.63	0.20, 1.15	.003**	0.75	-0.09, 1.75	.08	-1.81	-3.33, -0.60	.002**
- ADE	0.12	-0.66, 0.91	.75	-0.53	-2.38, 1.29	.58	0.44	-2.26, 3.23	.76
- Total effect	.75	-0.03, 1.54	.06	0.22	-1.21, 1.61	.73	.97	-10.72, 12.81	.28
DA									
- ACME	1.18	0.28, 2.43	.006**	1.47	0.00, 3.34	.05*	-3.29	-6.33, -0.96	.003**
- ADE	0.68	-0.80, 2.16	.36	-1.05	-4.28, 2.23	.52	-0.59	-5.70, 4.38	.82
- Total effect	1.86	0.33, 3.44	.02*	0.42	-2.29, 3.13	.77	-3.88	-8.91, 1.11	.12

Notes. CM = Concern over Mistakes; DA = Doubts about Actions; ACME = average causal mediation effects; ADE = average direct effects. *p < .05 **p <.01.

Table S5. Results from mediation analysis using DBAS as a mediator in the relationship between sleep discrepancy measures and selected subscales of perfectionism (*n* = 60, simulations = 10,000)

Sleep Discrepancy	SOL_D			WASO_D			TST_D		
Perfectionism	Estimate	95% *CI*	*p*	Estimate	95% *CI*	*p*	Estimate	95% *CI*	*p*
CM									
- ACME	0.00	-0.71, 0.72	.99	-0.10	-0.77, 0.53	.72	0.49	-1.31, 2.53	.59
- ADE	0.75	-0.51, 2.01	.24	0.33	-1.19, 1.81	.66	-1.84	-5.21, 1.55	.29
- Total effect	0.76	-0.12, 1.61	.09	0.23	-1.19, 1.65	.75	-1.35	-3.83, 1.15	.30
DA									
- ACME	-0.02	-1.43, 1.41	.96	-0.18	-1.45, 0.98	.74	1.07	-1.98, 4.91	.48
- ADE	1.90	-0.48, 4.31	.12	0.60	-1.99, 3.19	.66	-4.96	-11.03, 1.14	.11
- Total effect	1.88	0.20, 3.55	.03*	0.42	-2.26, 3.04	.75	-3.89	-9.12, 1.15	.14

Notes. CM = Concern over Mistakes; DA = Doubts about Actions; ACME = average causal mediation effects; ADE = average direct effects. *$p < .05$.

Table S6. Results from mediation analysis using DBAS subscale sleep Expectations (DBAS-E) as a mediator in the relationship between sleep discrepancy measures and selected subscales of perfectionism (*n* = 60, simulations = 10,000)

Sleep Discrepancy	SOL_D			WASO_D			TST_D		
Perfectionism	Estimate	95% *CI*	*p*	Estimate	95% *CI*	*p*	Estimate	95% *CI*	*p*
CM									
- ACME	-0.04	-0.37, 0.23	.80	-0.05	-0.42, 0.26	.76	0.11	-0.67, 1.06	.75
- ADE	0.79	-0.04, 1.63	.06	0.28	-1.13, 1.70	.69	-1.47	-3.80, 0.87	.22
- Total effect	0.75	-0.08, 1.65	.08	0.24	-1.22, 1.67	.75	-1.35	-3.76, 1.04	.26
DA									
- ACME	-0.32	-1.34, 0.29	.39	-0.37	-1.52, 0.30	.35	0.96	-0.84, 3.83	.35
- ADE	2.2	0.48, 3.96	.04*	0.80	-1.82, 3.38	.55	-4.82	-9.96, 0.33	.07
- Total effect	1.88	-1.52, 3.58	.02*	0.43	-2.21, 3.07	.75	-3.87	-9.04, 1.14	.13

Notes. CM = Concern over Mistakes; DA = Doubts about Actions; ACME = average causal mediation effects; ADE = average direct effects. *$p < .05$.

Table S7. Results from mediation analysis using DBAS subscale Worry about sleep (DBAS-W) as a mediator in the relationship between sleep discrepancy measures and selected subscales of perfectionism (*n* = 60, simulations = 10,000)

Sleep Discrepancy	SOL_D			WASO_D			TST_D		
Perfectionism	Estimate	95% *CI*	*p*	Estimate	95% *CI*	*p*	Estimate	95% *CI*	*p*
CM									
- ACME	0.05	-0.57, 0.68	.87	0.07	-0.61, 0.76	.83	0.18	-1.45, 1.94	.83
- ADE	0.70	-0.43, 1.83	.23	0.14	-1.54, 1.82	.86	-1.56	-4.93, 1.75	.35
- Total effect	0.75	-0.09, 1.59	.08	0.21	-1.20, 1.64	.77	-1.38	-3.81, 1.04	.27
DA									
- ACME	0.11	-0.76, 1.00	.78	0.12	-0.77, 1.08	.78	0.19	-1.94, 2.41	.84
- ADE	1.75	-0.18, 3.73	.08	0.30	-2.30, 2.88	.81	-4.04	-9.37, 1.46	.14
- Total effect	1.86	0.25, 3.45	.02*	0.42	-2.17, 2.95	.74	-3.84	-8.52, 0.93	.11

Notes. CM = Concern over Mistakes; DA = Doubts about Actions; ACME = average causal mediation effects; ADE = average direct effects. **p* < .05.

Table S8. Results from mediation analysis using DBAS subscale Consequences of insomnia (DBAS-C) as a mediator in the relationship between sleep discrepancy measures and selected subscales of perfectionism (*n* = 60, simulations = 10,000)

Sleep Discrepancy	SOL_D			WASO_D			TST_D		
Perfectionism	Estimate	95% *CI*	*p*	Estimate	95% *CI*	*p*	Estimate	95% *CI*	*p*
CM									
- ACME	0.06	-0.53, 0.66	.84	-0.20	-0.84, 0.32	.44	0.49	-0.89, 2.19	.47
- ADE	0.70	-0.44, 1.83	.24	0.43	-0.10, 1.87	.56	-1.85	-4.81, 1.12	.22
- Total effect	0.76	-0.11, 1.60	.09	0.23	-1.19, 1.65	.74	-1.35	-3.82, 1.04	.27
DA									
- ACME	0.29	-1.71, 1.78	.97	-0.62	-2.24, 0.79	.39	1.83	-1.83, 6.36	.32
- ADE	1.85	-0.92, 4.54	.19	1.04	-1.59, 3.67	.44	-5.71	-12.10, 0.57	.08
- Total effect	1.87	0.17, 3.50	.03*	0.42	-2.30, 2.97	.74	-3.88	-8.89, 1.21	.13

Notes. CM = Concern over Mistakes; DA = Doubts about Actions; ACME = average causal mediation effects; ADE = average direct effects. **p* < .05.

Table S9. Results from mediation analysis using DBAS subscale Medication (DBAS-M) as a mediator in the relationship between sleep discrepancy measures and selected subscales of perfectionism (*n* = 60, simulations = 10,000)

Sleep Discrepancy	SOL_D			WASO_D			TST_D		
Perfectionism	Estimate	95% *CI*	*p*	Estimate	95% *CI*	*p*	Estimate	95% *CI*	*p*
CM									
- ACME	0.05	-0.34, 0.50	.78	0.10	-0.24, 0.54	.56	0.15	-0.77, 1.24	.73
- ADE	0.71	-0.25, 1.69	.15	0.13	-1.25, 1.52	.85	-1.52	-4.27, 1.15	.28
- Total effect	0.76	-0.04, 1.58	.07	0.23	-1.16, 1.65	.75	-1.37	-3.90, 1.10	.27
DA									
- ACME	0.09	-0.49, 0.85	.78	0.14	-0.43, 0.95	.66	0.19	-1.18, 1.86	.79
- ADE	1.79	0.03, 3.54	.05*	0.27	-2.15, 2.68	.83	-4.06	-8.99, 0.92	.11
- Total effect	1.88	0.27, 3.46	.02*	0.41	-2.17, 3.04	.76	-3.87	-8.63, 0.94	.11

Notes. CM = Concern over Mistakes; DA = Doubts about Actions; ACME = average causal mediation effects; ADE = average direct effects. *$p < .05$.
